# The Alleged Vaccinal Syphilis in the 16th Ill. Infantry

**Published:** 1872-02-15

**Authors:** 


### Correspondence.

### THE ALLEGED VACCINAL SYPHILIS IN THE 16TH ILL. INFANTRY.

DOCTOR DAVIS:

*My Dear Sir* — I have heard several times during the past few years, that the 16th Ill. Infantry, during the war, were infected with syphilis from vaccination, and I determined to find out definitely about the matter, as soon as I could ascertain any proper and reliable authority to apply to. I hand you herewith two letters from Dr. Louis Watson, late Medical Director of the U. S. forces at Nashville, Tenn., under whose notice these cases were brought, and he certainly seems to know whereof he speaks. You can use the correspondence if you think it of any interest to your readers. Respectfully yours,

CHAS. GILMAN SMITH, M.D.

100 16th st., Feb. 10, '72.

---

TOPEKA, KAN., Jan. 18, '72.

CHAS. GILMAN SMITH, M.D.:

*Dear Sir* — Your letter of the 6th inst., addressed to Quincy, has been forwarded and is just received by me. You inform me that you "have heard that the 16th Ill. Inf. and some other regiments with whom I was connected during the war, were infected with syphilis from vaccination," and inquire "if this is really so?"

*Certainly not.* Such a report, whether originating from privates, officers, or even medical officers, has no foundation in fact. While Med. Dir. of Post forces at Nashville, in 1863, some of the members of the 2d Reg. Middle Tenn. Inf. were vaccinated from a *scab* obtained from the Med. Purveyor there. Some of these vaccinations were *followed* by "bad ulcers," not all of the same character, and which were more or less difficult to heal. Some were large, with destruction of cellular tissue beneath the cutis, but no more syphilitic than hospital grangrene.

Some of the 16th Ill. Inf. at Rossville, Ga., had ulcers following vaccination. The Reg't was then in charge of the Ass't Surg., Dr. Truat, a young man; not a graduate in medicine, still, a very worthy, careful, and judicious physician, and who, withal, had an earnest desire to improve himself in his profession. He was much perplexed in the treatment of the cases, and much annoyed by the declaration of certain medical officers of the Brigade, that "pox" had been communicated by vaccination. In consequence he corresponded with me, receiving my opinion and advice. He kept notes of cases and reported them to me.

Many of the Med. officers among the Volunteers were deficient, not only in education, but in experience. Some knew very little of the progress of a proper vaccine pustule. Many knew *less* about syphilis, whether primary, secondary, or tertiary, and their opinions, even if honestly given, were of no value. There were others whose education and character, or standing, ought to give weight to their opinions, who pronounced these ulcers syphilitic. But for the most part they were off-hand opinions, given without due examination and attention to the progress and *termination*. I am firm in the belief that there was no syphilis in any of these cases; though my opinion, to begin with, was that *syphilis* was not communicable by vaccination (indeed not by inoculation, except from primary ulcer). I do not deny that a vitiated ulcer may be produced, but that such ulcer is SYPHILIS; syphilis being a specific disease and having laws directing its progress, etc. Still I think I was competent to form, from my observations, a reasonably correct opinion.

My reply is unnecessarily long. It might be much longer by my going into statement of cases. But you have my answer. You can use anything herein in any manner you see fit; but as this letter is hastily written you should treat it accordingly.

Very respectfully,

LOUIS WATSON, M.D.

P. S.— If you can conveniently obtain some genuine vaccine virus to send me I would be much obliged for the same. A *recent* crust will answer my purpose. I am disposed to object to *revaccinations*, mainly for the reason that the profession in general do not make distinction in selecting and distributing virus — using lymph or crust obtained from pustules arising from *re*-vaccinations — and from this cause I believe it happens that vaccination is not so protective as formerly. One *good take* of the *true Jennerian virus* being sufficient for a lifetime as far as respects all practical purposes. Nearly, if not quite, all of the bad cases occurring in the 16th Ill. Reg't were revaccinations ! ! L. W.

---

Dr. Louis Watson, Topeka, Kan.:

*My Dear Doctor* — I do not agree with you that syphilis can never be conveyed by vaccination, though I am well aware that it is not so conveyed in one out of ten thousand of the cases so reported among people generally. It happens, I think, occasionally, when in vaccinating with the scab, taken from a syphilitic subject, some of the pus or dried blood on the scab is inserted with the true vaccine matter. I have never seen anything of the kind myself, but I think there is good authority for such a statement. If I had not lost my entire library by the fire, I could give you a good many references to such authority. I recall only one now. In the article on Vaccinia, in "Lawrence Smith on Children," you will find as eminent and trustworthy an observer as Dr. Alonzo Clark, of New York, quoted as having seen such cases.

With regard to the communication of syphilis from anything but the primary ulcer, which you allude to casually, I think all the authorities agree that it can be communicated from secondary manifestations, more especially from mucous patches, which, from their profuse secretion, seem to be one of the most common sources of infection.

But this is not what I wished to speak about particularly. I only wished to ask if you had any opportunity to watch these army cases afterwards, more especially after two months subsequent to the vaccination? Did any of these subjects who had the ulcers, have general eruption on the skin, sore throat, coming out of the hair, iritis, or any of the well-known manifestations of syphilitic infection, coming on two or three months after they were vaccinated? If you could give something more explicit on these points — something of the subsequent history of the patients, I should be very glad to have it.

Yours respectfully,

Charles G. Smith, M.D.

100 16th st.

---

Topeka, K's, Feb. 5th, '72.

C. Gilman Smith, M.D.:

*Dear Sir* — I have delayed replying to your letter of Jan. 24th without an adequate excuse. In reply to your questions in regard to the appearance of secondary or tertiary syphilis subsequent to the vaccinations alluded to, I would state that I cannot report as to the Tenn. Reg't, but as to the 16th Ill., from my own observations for the period of one year, (and of some of the subjects up to last April,) and upon the authority of Dr. Truat, the Ass't Surg., who had charge of the Reg't up to Nov., 1864, I am positive that no such symptoms occurred, which could with any sort of propriety be attributed to those vaccinations. We had *very little* true syphilis in our portion of the army, gonorrhœa and chancroids being the principal venereal diseases. The question whether syphilis could be communicated or transmitted by vaccination was discussed in the Adams Co. Med. Society about eighteen months ago, at which time Dr. H. W. Kendall of Quincy was to have taken the affirmative, he claiming that he had seen it in the army. He was surgeon of the 50th Ill. But, as he did not appear at the discussion, appointed for two meetings at his request, I do not know what the character of the evidence he intended to submit might have been.

From the various sources whence the vaccine material was obtained, and the fact that true vaccinia as well as these bad ulcers, suppurations, etc., would follow its insertion, I infer that the bad results were the consequence of a cachexia existing in the subjects. What the cachexia may have been I do n't know, but I suspect scurvy. There were some half dozen unequivocal cases in the 16th at that time, and Dr. Truat told me that full half of those up at "morning call" had swollen and tender gums. If we had reports of similar results of vaccination on shipboard, during a long voyage, I might be confirmed in this opinion. As *all* the cases in the 16th were revaccinations, I do not know but this circumstance might have something to do with it. On the subject of the transmission of disease by vaccination, there was a prolonged discussion in the Imperial Academy of Med. of Paris during the fall of 1870, the result of which is alluded to in the "N. Y. Med. Journal" of Jan., 1871, quoting from "Le Union Medicale" of Sept. 9th, '70. The decision was in the negative.

I thank you for the reliable vaccine virus you sent me. Very respectfully yours,

L. Watson, M.D.

